# The degree of microsatellite instability predicts response to PD-1 blockade immunotherapy in mismatch repair-deficient/microsatellite instability-high colorectal cancers

**DOI:** 10.1186/s40164-020-00193-z

**Published:** 2021-01-04

**Authors:** Qiao-Xuan Wang, Chun-Hua Qu, Yuan-Hong Gao, Pei-Rong Ding, Jing-Ping Yun, Dan Xie, Mu-Yan Cai

**Affiliations:** 1 Collaborative Innovation Center for Cancer Medicine, State Key Laboratory of Oncology in South China, Sun Yat-Sen University Cancer Center, No. 651, Dongfeng Road East, Guangzhou, 510060 China; 2 Department of Radiation Oncology, Sun Yat-Sen University Cancer Center, Guangzhou, 510060 China; 3 Department of Pathology, Sun Yat-Sen University Cancer Center, Guangzhou, 510060 China; 4 Department of Colorectal Surgery, Sun Yat-Sen University Cancer Center, Guangzhou, 510060 China

**Keywords:** dMMR/MSI-H, Anti-PD-1 immunotherapy, Colorectal cancer

## Abstract

The development of programmed cell death-1 inhibitor (PD-1) has shed light on the treatment of tumors with deficiencies in DNA mismatch repair system or microsatellite instability (dMMR/MSI). However, predicting the subset in this group that will benefit from PD-1 blockade remains a challenge. In this study, we aimed to investigate the relationship between the degree of microsatellite instability and the responses to anti-PD-1 immunotherapy. 33 patients with colorectal adenocarcinoma who had a known MSI status and received anti-PD-1 immunotherapy were included. PCR results for MSI of the whole cohort were collected and treatment response was evaluated. Our data indicated that objective response rate (ORR) in instability-high group (instability loci ≥ 3) was significantly higher than ORR in instability-intermediate group (13/16 versus 6/17, *P* = 0.008). Besides, patients in instability-high group had significant longer progression-free survival (log-rank test, *P* = 0.004), and a significant increase in T lymphocyte infiltration and cytolytic activity in tumors. Future study might implement the intensity of microsatellite instability for more delicate selection for anti-PD-1 therapy in patient with dMMR/MSI-H tumors.

## To the Editor

Defects in DNA mismatch repair (dMMR) promote a frequent insertion and/or deletion hypermutable state in nucleotide repeats regions termed microsatellite instability-high (MSI-H) [1]. Colorectal cancers (CRCs) with dMMR/MSI-H have favorable response to the programmed cell death-1 (PD-1) blockade immunotherapy [2]. However, there are still 45–70% of such tumors which do not respond to immune checkpoint blockade, so predicting the subset that will benefit from PD-1 blockade remains a challenge [3–5]. In the current study, we aimed to evaluate whether the degree of microsatellite instability can predict the diversity of responses to anti-PD-1 immunotherapy in dMMR/MSI-H colorectal cancers.

## Methods

Patients’ data were collected from a prospectively maintained database in Sun Yat-sen University Cancer Center, Guangzhou, China. Inclusion criteria were as follows: (1) pathologically confirmed colorectal adenocarcinoma; (2) a known MSI status; (3) received at least one dose of anti-PD-1 therapy. Patients with tumors demonstrating no instability loci (microsatellite stable, MSS) were excluded, except for those whose tumor was proved to be dMMR. MSI or dMMR status was prospectively determined using the American National Cancer Institute-recommended Polymerase Chain Reaction (PCR) for MSI or immunohistochemistry (IHC) for dMMR. IHC analyses of CD3 and CD8 were performed using the standard methods on pretreatment specimens. The ethical committees in our center approved this study procedure and waive the necessity of informed consult.

Treatment response was evaluated every two cycles of treatment according to RECIST v1.1. Objective response rate (ORR) was defined as the portion of patients with complete response (CR) or partial response (PR). Progression-free survival (PFS) was calculated from the date of initial anti-PD-1 treatment to either the date of the first progression or death due to CRC. ORRs between groups were compared by χ2 test. PFS was estimated using the Kaplan–Meier method and compared between groups with the log-rank test. Multivariate analyses were performed using the Cox proportional hazards model.

## Results

Thirty-three patients were included in the study, with eighteen men and a median age of 45 years (range, 19 to 67). Baseline characteristics were shown in Table 1. Two patients were diagnosed with stage II disease (one local recurrent), seven patients were with stage III disease, while 24 patients were with stage IV disease. Among them, nineteen patients had received ≥ 2 lines of prior systemic therapies before anti-PD-1 immunotherapy. Thirteen patients were treated with a single-agent anti-PD-1 antibody, and the median cycles of therapy given were 8 (range 1–31).

**Table 1:** Baseline characteristics of patients according to the degrees of MSI

Variables	Instability loci < 3	Instability loci ≥ 3	*P* value
n	17	16	
Age-median (range), year	45.0 (19.0–64.0)	45.5 (30.0–67.0)	0.783
Sex-n			0.112
Male	7	11	
Female	10	5	
Tumor stage-n			0.619
II/III	4	5	
IV	13	11	
Lines of therapy-n			0.119
First line	5	9	
Second or late line	12	7	
Combined with chemotherapy-n			0.055
Yes	13	7	
No	4	9	

As shown in Table 2, we found that three patients identified as d-MMR but with microsatellite stability (MSS) disease had no response to anti-PD-1 treatment. By contrast, all patients with five instability loci achieved PR or CR (Additional file 1: Figure S1). To investigate the relationship between the degree of MSI and responses to anti-PD-1 therapy, we classified the patients into instability-intermediate (instability loci < 3) and instability-high subgroups (instability loci ≥ 3) according to the degrees of MSI. Baseline characteristics were comparable between the two groups (Table 1). However, ORR in instability-high group was significantly higher than ORR in instability-intermediate group (13/16 versus 6/17, *P* = 0.008).

**Table 2:** Treatment response and ORR of patients stratified by the number of instability loci

Number of instability loci	Number of cases	CR^*^	PR	SD	PD	ORR
0	3	0	0	2	1	0
1	5	0	0	2	3	0
2	9	2	4	1	2	0.67
3	9	5	2	1	1	0.78
4	4	2	1	1	0	0.75
5	3	1	2	0	0	1

*CR* complete response, *PR* partial response, *SD* stable disease, *PD* progressive disease, *ORR* objective response rate

^*^For patients who underwent surgical resection and had a pathological confirmed complete response, treatment response was recorded as CR

**Fig. 1:**
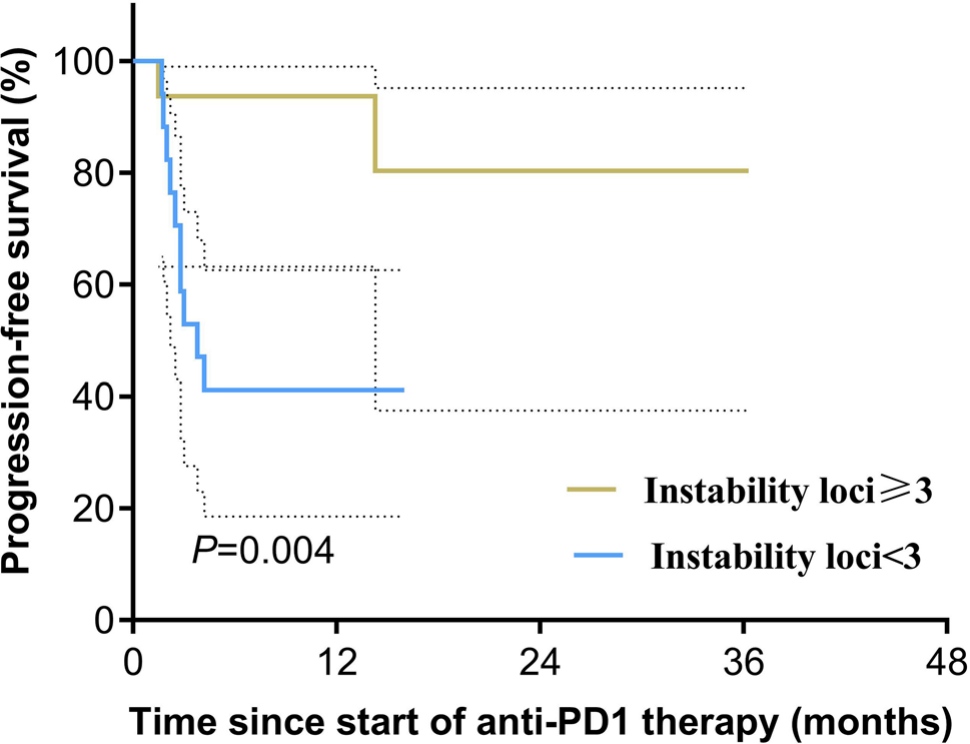
Progression-free survival of the two groups according to the degree of MSI. Survival was analyzed using the log-rank test. Dashed lines are 95% confidence intervals

During a median follow-up of 11.2 months (range, 2.0–36.3), twelve patients had the disease progression. All patients remained alive at the wrighting of this article. Univariable analysis showed that the degree of microsatellite instability was associated with PFS after anti-PD-1 immunotherapy (Additional file 1: Table S1). Patients in instability-high subgroup have significant longer PFS (log-rank test, *P* = 0.004, Fig. 1). After excluding the confounding effects of sex, age and combined treatment by multivariate Cox proportional hazard model, instability-high was demonstrated to be an independent predictor for the longer PFS (HR = 0.136 [0.024–0.781], *P* = 0.025; Additional file 1: Table S2). Further univariable analysis of the location of the MSI loci showed that mutation in the loci of BAT25 and BAT26 were associated with longer PFS (Additional file 1: Table S3). To exclude the confounding effect of the number of instability loci, we did the multivariate cox regression for PFS, and BAT25 retains its predictive capability (HR = 0.037 [0.002–0.571], *P* = 0.018; Additional file 1: Table S4).

**Fig. 2:**
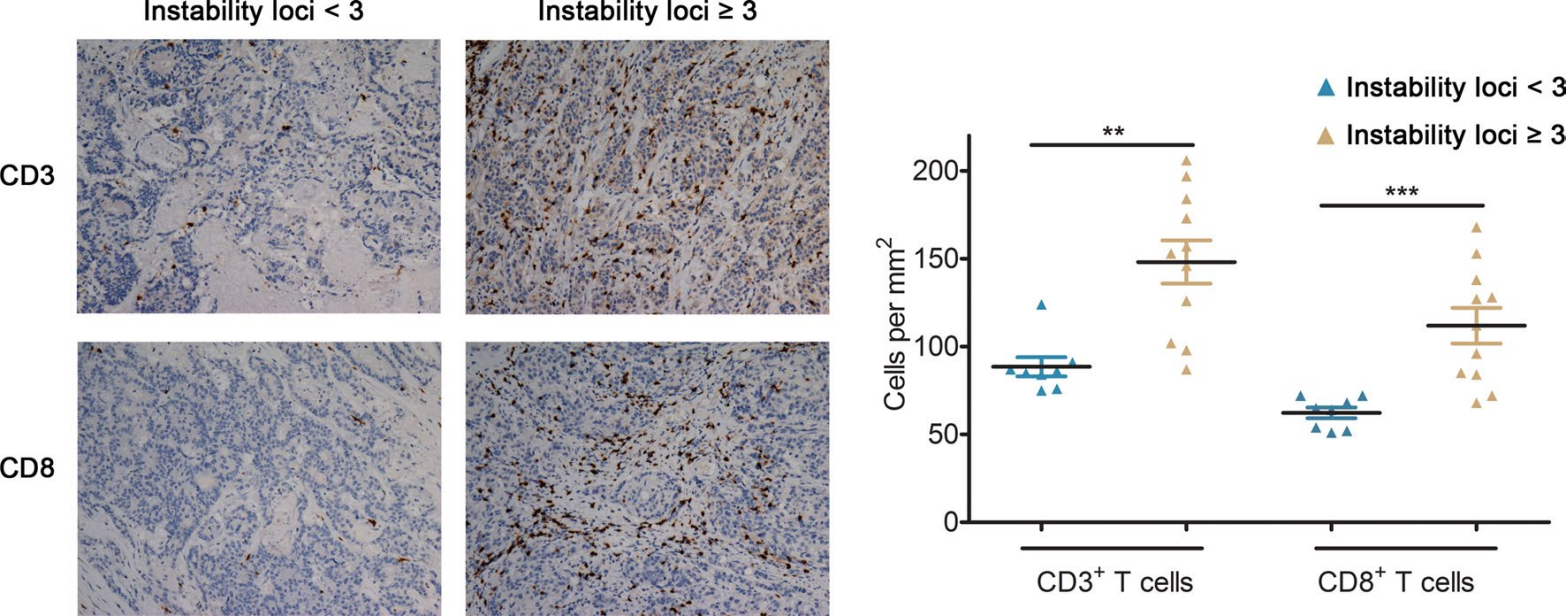
The degrees of microsatellite instability in relation to T cell infiltration in dMMR/MSI-H CRC. Representative immunohistochemical images of pretreatment tumor specimens stained with CD3 and CD8. Images are shown at ×20 with a 100-μM scale bar in each image

IHC analyses of CD3 and CD8 were performed and the number of CD3+ and CD8+ T cell infiltration per mm^2^ was counted in each group. A significant increase in CD3+ and CD8+ T lymphocyte infiltration and cytolytic activity were also found in tumors in instability-high subgroup (Fig. 2).

## Discussion

Our study indicated that the degree of microsatellite instability could predict a patient’s response to anti-PD-1 immunotherapy, and was an independent predictor for PFS in dMMR/MSI-H CRCs. This is in line with the recent finding in mouse models of microsatellite instability [6]. Underling mechanisms might involve the increased tumors immunogenicity and lymphocytic infiltration, as MSI-H reflects a genome-wide instability which eventually results in high mutational burden.

The main limitation of this study is the small sample size. But our data suggest that responds to anti-PD-1 are substantially diverse within dMMR tumors and that it highlights the possibility of more delicate selection for anti-PD-1 therapy in patients with dMMR/MSI-H.

## Supplementary Information

**Additional file 1:** **Figure S1. **The intensity of instability loci and the patient’s response to anti-PD-1 treatment. A. The representative PCR result of the colorectal cancer patient (case 6) with five microsatellite instability loci. B. The representative MRI images of the patient (case 6) showing a sigmoid colon mass before and after anti-PD-1 immunotherapy. **Table S1.** Univariate analyses of the prognostic factors for progression-free survival of the cohort (N = 33). **Table S2.** Multivariate Cox regression analyses of the prognostic factors for progression-free survival of the whole cohort (N = 33). **Table S3.** Univariate analyses of the location of the MSI loci for progression-free survival of the cohort (N = 33). **Table S4.** Multivariate Cox regression analyses for progression-free survival of the whole cohort (N = 33).

## Data Availability

The datasets analysed during the current study are available from the corresponding author on reasonable request.

## Abbreviations

dMMR: Defects in DNA mismatch repair
MSI-H: Microsatellite instability-high
CRCs: Colorectal cancers
PD-1: Programmed cell death-1
PCR: Polymerase Chain Reaction
IHC: Immunohistochemistry
ORR: Objective response rate
CR: Complete response
PR: Partial response
PFS: Progression-free survival
