# Supplementary material for: The degree of microsatellite instability predicts response to PD-1 blockade immunotherapy in mismatch repair-deficient/microsatellite instability-high colorectal cancers
Source: Exp Hematol Oncol. 2021 Jan 4;10:2. doi: 10.1186/s40164-020-00193-z (PMC7784284; doi:10.1186/s40164-020-00193-z)
Supplement: Supplementary file 1 — Additional file 1: Figure S1. The intensity of instability loci and the patient’s response to anti-PD-1 treatment. A. The representative PCR result of the colorectal cancer patient (case 6) with five microsatellite instability loci. B. The representative MRI images of the patient (case 6) showing a sigmoid colon mass before and after anti-PD-1 immunotherapy. Table S1. Univariate analyses of the prognostic factors for progression-free survival of the cohort (N = 33). Table S2. Multivariate Cox regression analyses of the prognostic factors for progression-free survival of the whole cohort (N = 33). Table S3. Univariate analyses of the location of the MSI loci for progression-free survival of the cohort (N = 33). Table S4. Multivariate Cox regression analyses for progression-free survival of the whole cohort (N = 33). [file 40164_2020_193_MOESM1_ESM.docx]

**Figure S1. The intensity of instability loci and the patient’s response to anti-PD-1 treatment.**

1. The representative PCR result of the colorectal cancer patient (case 6) with five microsatellite instability loci. B. The representative MRI images of the patient (case 6) showing a sigmoid colon mass before and after anti-PD-1 immunotherapy.


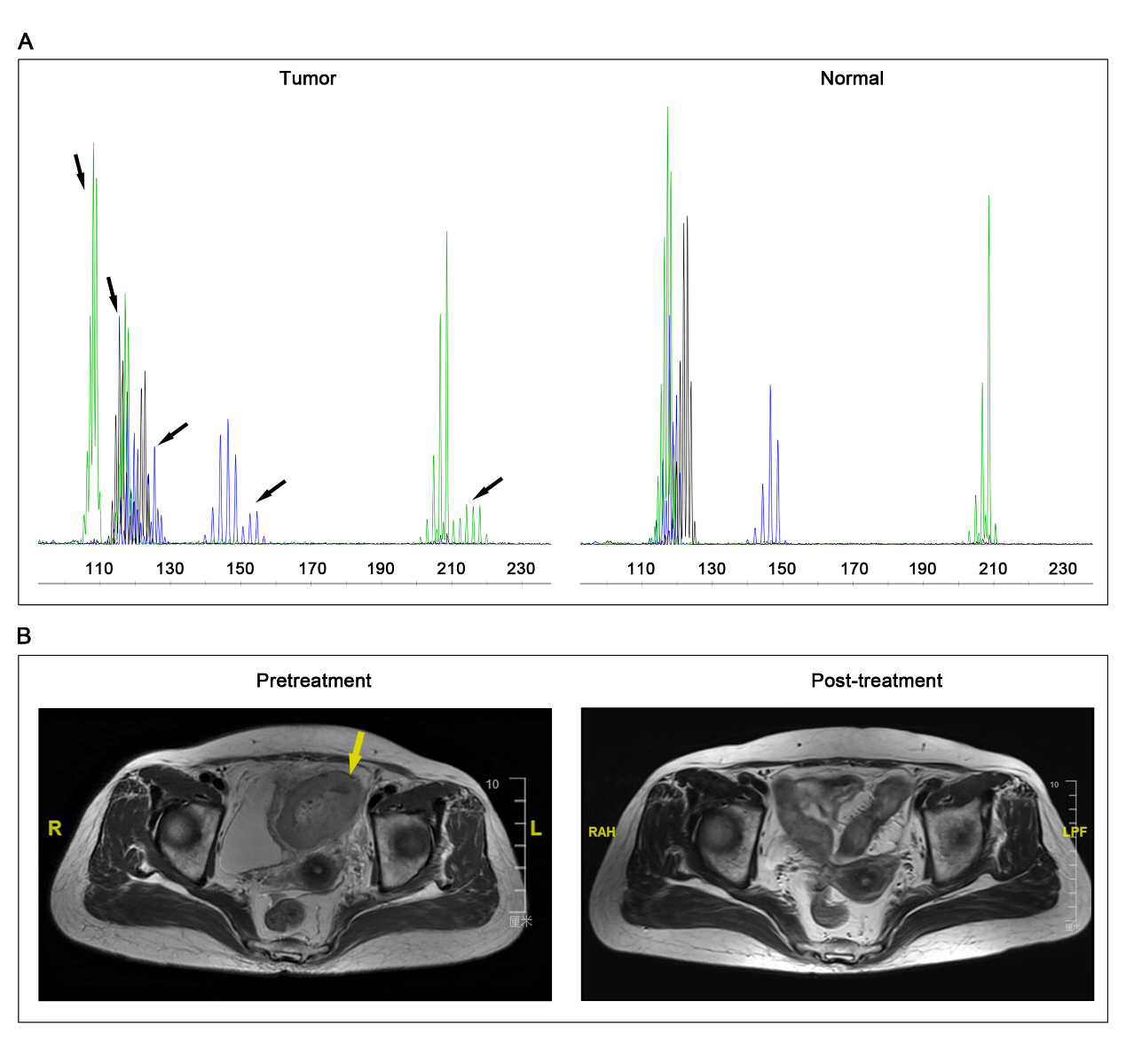


**Table S1. Univariate analyses of the prognostic factors for progression-free survival of the cohort (N=33).**

| Variable | HR（95% CI） | P-value |
| --- | --- | --- |
| Age, y (<60: ≥60) | 0.341 (0.044-2.648) | 0.303 |
| Sex (male: female) | 1.972 (0.625-6.227) | 0.247 |
| Tumor stage (II/III: IV) | 37.66 (0.211-6721.451) | 0.170 |
| PD-1 blockade as first line of therapy (yes: no) | 65.998 (0.62-7022.083) | 0.079 |
| PD-1 monotherapy (yes: no) | 2.214 (0.598-8.191) | 0.234 |
| The degree of MSI (instability loci<3: ≥ 3) | 0.132 (0.027-0.639) | 0.012 |

**Table S2. Multivariate Cox regression analyses of the prognostic factors for progression-free survival of the whole cohort (N=33).**

| Variable | HR（95%CI） | P-value |
| --- | --- | --- |
| Age, y | 1.015 (0.976-1.055) | 0.456 |
| Sex (male: female) | 1.413 (0.443-4.614) | 0.567 |
| PD-1 monotherapy (yes: no) | 1.014 (0.243-4.228) | 0.985 |
| The degree of MSI (instability loci<3: ≥ 3) | 0.136 (0.024-0.781) | 0.025 |

**Table S3. Univariate analyses of the location of the MSI loci for progression-free survival of the cohort (N=33).**

|  | PFS events | | Univariable analysis for PFS | | |
| --- | --- | --- | --- | --- | --- |
| Instability loci | mutant cases | wide-type cases | HR（mutant：wide-type） | | *P* value |
| D5S346 | 8/16 | 4/17 | 0.390 （0.117-1.303） | 0.126 | |
| BAT25 | 2/21 | 10/12 | 16.762 （3.590-78.269） | 0.000 | |
| BAT26 | 4/24 | 8/9 | 11.890 （3.072-46.010） | 0.000 | |
| D17S250 | 1/8 | 11/25 | 3.715 （0.479-28.8） | 0.209 | |
| D2S123 | 2/12 | 10/21 | 3.760 （0.813-17.385） | 0.090 | |

**Table S4. Multivariate Cox regression analyses for progression-free survival of the whole cohort (N=33).**

| Variable | HR（95%CI） | *P* value |
| --- | --- | --- |
| BAT25 | 0.037 (0.002-0.571) | 0.018 |
| BAT26 | 0.151 (0.015-1.510) | 0.108 |
| Number of instability loci | 1.946 (0.665-5.691) | 0.224 |
